# Transcatheter and surgical aortic valve replacement in patients with left ventricular dysfunction

**DOI:** 10.1186/s13019-022-02061-9

**Published:** 2022-12-18

**Authors:** Maina P. Jalava, Mikko Savontaus, Tuomas Ahvenvaara, Teemu Laakso, Marko Virtanen, Matti Niemelä, Tuomas Tauriainen, Pasi Maaranen, Annastiina Husso, Eve Kinnunen, Sebastian Dahlbacka, Jussi Jaakkola, Stefano Rosato, Paola D’Errigo, Mika Laine, Timo Mäkikallio, Peter Raivio, Markku Eskola, Antti Valtola, Tatu Juvonen, Fausto Biancari, Juhani Airaksinen, Vesa Anttila

**Affiliations:** 1 Heart Centre, Turku University Hospital and University of Turku, P. O. Box 52, 20521 Turku, Finland; 2 Department of Surgery, Oulu University Hospital and University of Oulu, Oulu, Finland; 3 Heart Center, Helsinki University Hospital, Helsinki, Finland; 4 Heart Hospital, Tampere University Hospital and University of Tampere, Tampere, Finland; 5 Department of Internal Medicine, Oulu University Hospital, Oulu, Finland; 6 Heart Center, Kuopio University Hospital, Kuopio, Finland; 7 National Centre of Global Health, Istituto Superiore di Sanità, Rome, Italy; 8 Clinica Montevergine, GVM Care and Research, Mercogliano, Italy

**Keywords:** Transcatheter aortic valve replacement, TAVR, Surgical aortic valve replacement, SAVR, Aortic stenosis, AS, Left ventricular ejection fraction, Left ventricular dysfunction, Heart failure

## Abstract

**Background:**

Patients with severe aortic stenosis and left ventricular systolic dysfunction have a poor prognosis, and this may result in inferior survival also after aortic valve replacement. The outcomes of transcatheter and surgical aortic valve replacement were investigated in this comparative analysis.

**Methods:**

The retrospective nationwide FinnValve registry included data on patients who underwent transcatheter or surgical aortic valve replacement with a bioprosthesis for severe aortic stenosis. Propensity score matching was performed to adjust the outcomes for baseline covariates of patients with reduced (≤ 50%) left ventricular ejection fraction.

**Results:**

Within the unselected, consecutive 6463 patients included in the registry, the prevalence of reduced ejection fraction was 20.8% (876 patients) in the surgical cohort and 27.7% (452 patients) in the transcatheter cohort. Reduced left ventricular ejection fraction was associated with decreased survival (adjusted hazards ratio 1.215, 95%CI 1.067–1.385) after a mean follow-up of 3.6 years. Among 255 propensity score matched pairs, 30-day mortality was 3.1% after transcatheter and 7.8% after surgical intervention (*p* = 0.038). One-year and 4-year survival were 87.5% and 65.9% after transcatheter intervention and 83.9% and 69.6% after surgical intervention (restricted mean survival time ratio, 1.002, 95%CI 0.929–1.080, *p* = 0.964), respectively.

**Conclusions:**

Reduced left ventricular ejection fraction was associated with increased morbidity and mortality after surgical and transcatheter aortic valve replacement. Thirty-day mortality was higher after surgery, but intermediate-term survival was comparable to transcatheter intervention.

*Trial registration* The FinnValve registry ClinicalTrials.gov Identifier: NCT03385915.

**Supplementary Information:**

The online version contains supplementary material available at 10.1186/s13019-022-02061-9.

## Introduction

The prevalence of aortic stenosis (AS) and left ventricular (LV) dysfunction is increasing due to the aging population [1, 2]. Patients with AS and associated LV systolic dysfunction have a poor prognosis, even if they are asymptomatic [3]. This condition may result in inferior survival even after aortic valve replacement [4]. The outcomes after both transcatheter aortic valve replacement (TAVR) and surgical aortic valve replacement (SAVR) have improved over the last decade [5], but the incidence of congestive heart failure and mortality after both interventions remains high among patients with LV dysfunction [4, 6]. The feasibility of TAVR is documented in AS patients with high surgical risk [7].

In patients with AS and reduced LV ejection fraction (LVEF) the optimal treatment modality choice is unclear. The purpose of this comparative analysis was to investigate the short- and intermediate-term outcome of this patient group treated with TAVR or SAVR in a nationwide patient cohort.

## Materials and methods

The FinnValve registry is a nationwide registry (ClinicalTrials.gov Identifier: NCT03385915) containing data from consecutive and unselected patients who underwent TAVR or SAVR with a bioprosthesis for severe AS at Finnish university hospitals from 2008 to 2017 [5]. Patients with AS with or without aortic valve regurgitation, aged > 18 years, and who underwent primary TAVR or SAVR with a bioprosthesis with or without concomitant coronary artery revascularization were included. Patients with prior TAVR or surgical intervention on the aortic valve, concomitant procedure on the ascending aorta and/or other heart valves or structures, TAVR or SAVR for isolated aortic valve regurgitation, and/or acute endocarditis were excluded. The Finnish Institute for Health and Welfare provided data on date and causes of mortality, which is routinely collected from death certificates issued by physicians. The last date of follow-up was December 31, 2018. Secondary early outcomes were recorded during the index hospitalization. The echocardiographic assessments were made by experienced cardiologists and/or cardiac anesthesiologists depending on the local institute practice. The pre- and perioperative timing for echocardiographic assessment varied between the cohorts and institutions. The exact method for determining LVEF for each patient was not captured to the FinnValve registry data.

### Definition criteria of baseline risk factors

Severe AS was defined according to current guidelines [8, 9] by echocardiography. LV dysfunction was defined as LVEF ≤ 50% according to the EuroSCORE II criteria [10]. LVEF ≤ 50% was further dichotomized in to LVEF 30–50% and LVEF < 30% groups. Baseline variables were defined according to the EuroSCORE II criteria. The operative risk was stratified according to the EuroSCORE II and STS [11] risk scores. Frailty was defined according to the Geriatric Status Scale (GSS) [12] grades 2–3. Severe coronary artery disease was defined as any stenosis ≥ 50% of the main coronary branches. Critical preoperative state was defined as ventricular tachycardia or ventricular fibrillation or aborted sudden death, preoperative cardiac massage, preoperative ventilation before anesthetic room, preoperative inotropes or intra-aortic balloon pump (IABP) insertion and/or preoperative acute renal failure. Patients with critical preoperative state were included in patients with recent acute heart failure.

### Outcome measures

The primary outcomes were 30-day, 1-year and 4-year survival. The secondary outcomes during the index hospitalization were stroke, use intra-aortic balloon pump (IABP) and/or extracorporeal membrane oxygenation (ECMO), red blood cell (RBC) transfusions, transfusion of > 4 units of RBC and/or re-sternotomy for bleeding [13], and/or transfusion of > 4 units of RBC and/or any reoperation for intrathoracic or peripheral bleeding, major and life threatening bleeding [13], major vascular complication [13], moderate-to-severe paravalvular regurgitation, implantation of permanent pacemaker, acute kidney injury (AKI) and postoperative length of index hospital stay.

### Definition of outcomes

Major vascular complications were defined according to Valve Academic Research Consortium-2 consensus document (VARC-2) criteria [13]. Stroke was defined as any neurological deficit lasting ≥ 24 h with a new brain infarct or hemorrhage at neuroimaging, or a neurological deficit resulting in death. Major and life-threatening bleeding were defined according to VARC-2 criteria excluding perioperative decline in the hemoglobin levels because a perioperative decrease of hemoglobin levels is observed in most of patients undergoing SAVR and this does not always reflect a condition of major perioperative blood loss. European Coronary Artery Bypass Grafting (E-CABG) bleeding grades 2–3 was defined as transfusion of > 4 units of red blood cells and/or resternotomy for bleeding [14]. AKI was defined according to the Kidney Disease: Improving Global Outcomes (KDIGO) classification criteria [15]. Cardiac death was defined as any death occurring from coronary artery disease, valvular heart disease, heart failure, conduction disturbances, endocarditis, sudden cardiac death or death during the index procedure.

### Statistical analysis

Statistical analysis was performed using SAS statistical package, version 9.2 (SAS Institute Inc, Cary, NC), SPSS v. 26.0 statistical software (IBM Corporation, New York, USA) and Stata v. 15.0 (SAS Institute Inc., Cary, NC, USA).

Continuous variables were summarized as mean and standard deviation and categorical variables as counts and percentages. Normal distribution of continuous variables was assessed with the Shapiro–Wilk’s test. In the unmatched main cohort, Chi-squared test, Fisher’s exact test and Mann–Whitney *U*-test were used for univariable analysis. The Kaplan–Meier method was used to estimate late survival. Outcomes were adjusted in logistic regression and Cox proportional hazards models, using the enter mode and including the following covariates: age, gender, body mass index, glomerular filtration estimated according to the MDRD equation (eGFR), LVEF ≤ 50%, diabetes, dialysis, prior stroke, recent myocardial infarction, pulmonary disease, oxygen therapy, atrial fibrillation, extracardiac arteriopathy, frailty, recent acute heart failure, systolic pulmonary artery pressures, New York Heart Association (NYHA) class IV symptoms, urgency of the procedure, severe coronary artery disease, left main disease, number of diseased coronary arteries, prior cardiac surgery, prior percutaneous coronary intervention, planned concomitant revascularization, active malignancy, prior pacemaker, mitral regurgitation (mild, moderate and severe individually) and anemia. These regression analyses were performed separately for the unmatched TAVR and SAVR cohorts.

Patients with LVEF ≤ 50% were the subjects of a propensity score matching analysis comparing the outcomes after TAVR and SAVR. The propensity score was estimated using a non-parsimonious logistic regression model including the covariates as follows: age, gender, body mass index, anemia, eGFR, prior dialysis, diabetes, stroke and transient ischemic attack, pulmonary disease, oxygen therapy, extracardiac arteriopathy, porcelain aorta, atrial fibrillation, frailty, active malignancy, LVEF classes, systolic pulmonary artery pressure, mitral regurgitation, coronary artery disease, left main coronary stenosis, number of diseased coronary arteries, prior pacemaker, prior percutaneous coronary intervention, prior cardiac surgery, recent myocardial infarction, recent acute heart failure, NYHA class 4 symptoms, urgency, planned concomitant revascularization, EuroSCORE II and STS scores. One-to-one propensity score matching was performed employing the nearest neighbor method and a caliper width of 0.2, which was the 0.2 of the standard deviation of the logit of the propensity score, i.e. 1.068. To evaluate the balance between the matched groups, the t-test for paired samples for continuous variables and the McNemar test for dichotomous were used. Standardized differences < 0.10 were considered an acceptable imbalance between the groups. Baseline characteristics and early outcomes in the propensity score matched cohorts were evaluated using the paired t-test and the McNemar test. Differences in the long-term survival of matched pairs was evaluated by the Kaplan–Meier method. Since the proportional hazard assumption did not hold as assessed graphically and based on Schoenfeld’s residuals (global test: *p* = 0.080), the impact of treatment method on 4-year survival in propensity score-matched pairs was estimated using the restricted mean survival time (RMST) method. All tests were two-sided and *p* < 0.05 was set for statistical significance.

## Results

**Fig. 1:**
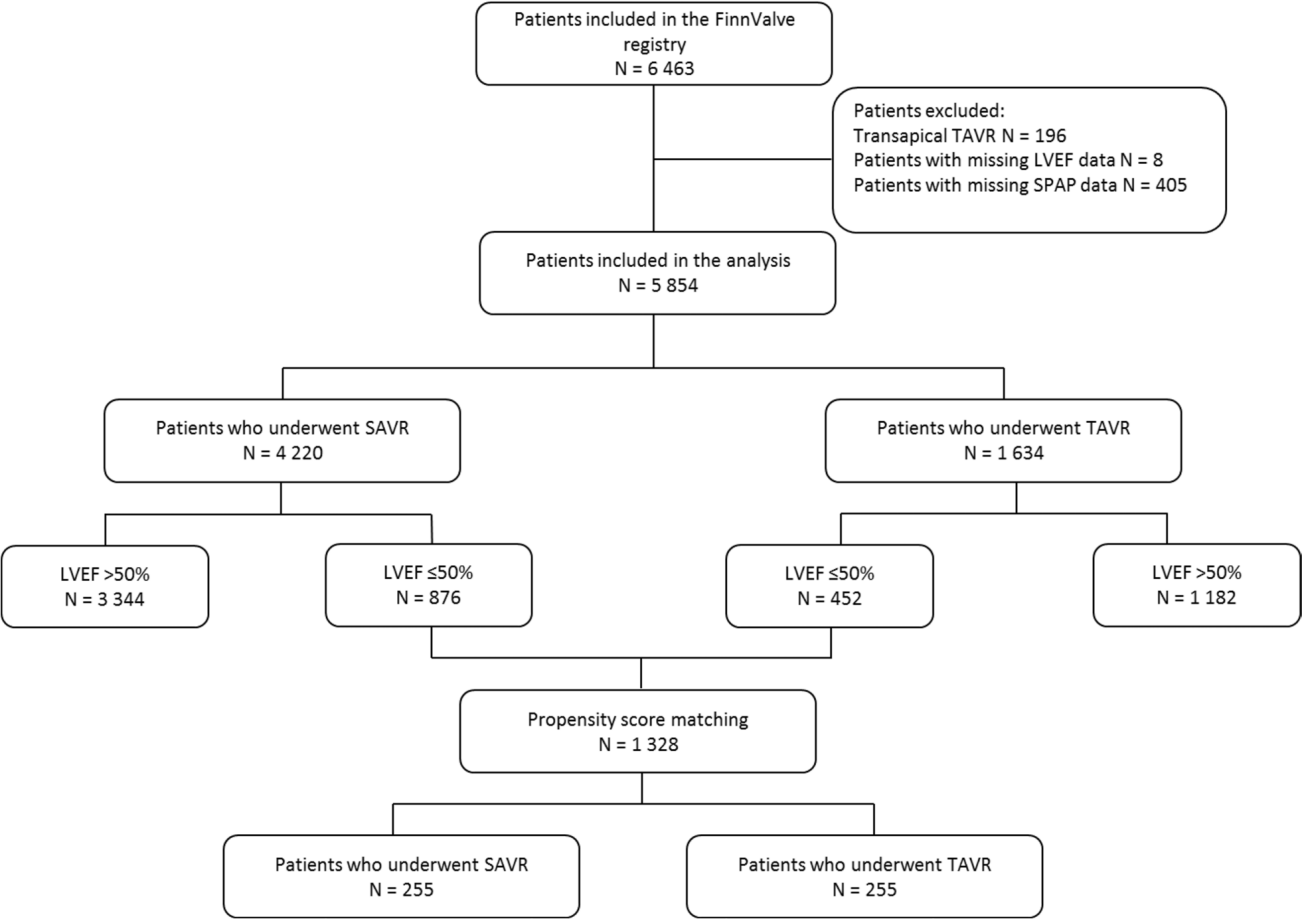
Study flowchart

The FinnValve registry includes data from 6463 patients who underwent TAVR or SAVR with bioprosthesis for severe AS. After excluding patients who underwent transapical TAVR and those without data on the LVEF and pulmonary artery pressures, 5854 patients were available for the present analysis (Fig. 1). The prevalence of LVEF ≤ 50% was 20.8% (876 patients) in the SAVR cohort and 27.7% (452 patients) in the TAVR cohort. However, among patients with LVEF ≤ 50%, TAVR was the most common procedure for AS since 2016 (Additional file 1: Fig. S1). The mean length of follow-up was 2.9 ± 1.8 years after TAVR and 4.4 ± 2.9 years after SAVR cohort.

The characteristics and outcomes of the main cohort are presented in Additional file 1: Tables S1 and S2. In the entire cohort, LVEF ≤ 50% was associated with decreased intermediate survival (adjusted HR 1.215, 95%CI 1.067–1.385). However, when adjusted for baseline variables, LVEF ≤ 50% was not associated with increased 30-day mortality after SAVR (OR 0.999, 95%CI 0.647–1.540, *p* = 1.000) or TAVR (OR 1.171, 95%CI 0.508–2.698, *p* = 0.71). The risk of death at intermediate follow-up was increased after SAVR (HR 1.238, 95%CI 1.060–1.445, *p* = 0.007), but not after TAVR (HR 1.080, 95%CI 0.840–1.388, *p* = 0.548). There was no difference in baseline LVEF levels between these procedures (Additional file 1: Table S3). Furthermore, the degree of reduction in LVEF did not affect survival in patients with LVEF ≤ 50% (Additional file 1: Table S4).

### *Propensity score matching analysis in patients with LVEF* ≤ *50%*

Propensity score matching resulted in 255 pairs with balanced baseline risk factors (Table 1). SAVR patients underwent more frequently planned concomitant coronary artery revascularization compared to TAVR patients (29.4 vs. 5.1%, *p* < 0.0001), despite similar prevalence (SAVR 36.5% vs. TAVR 38.4%) and severity of coronary artery disease (Table 1). Mean aortic valvular gradient was 46 ± 16 mmHg in TAVR patients and 46 ± 14 mmHg in SAVR patients (*p* = 0.848) (Table 1).

**Table 1:** Characteristics of propensity score matched patients with LVEF ≤ 50% undergoing surgical or transcatheter aortic valve replacement

Characteristics	SAVR 255 pts	TAVR 255 pts	Standardized difference	*p*-value
Age, years	79.8 ± 5.0	79.2 ± 7.3	0.037	0.690
Female	111 (43.5)	106 (41.6)	0.040	0.729
BMI, kg/m^2^	26.5 ± 4.6	26.7 ± 5.0	0.036	0.755
Anemia	137 (53.7)	132 (51.8)	0.039	0.718
eGFR, ml/min/1.73m2	65.3 ± 20.7	64.6 ± 23.8	0.034	0.770
Dialysis	3 (1.2)	3 (1.2)	0.000	1.000
Diabetes	69 (27.1)	73 (28.6)	0.035	0.762
Stroke	26 (10.2)	28 (11.0)	0.025	0.888
Pulmonary disease	60 (23.5)	58 (22.7)	0.019	0.920
Oxygen therapy	1 (0.4)	1 (0.4)	0.000	1.000
Extracardiac arteriopathy	47 (18.4)	40 (15.7)	0.073	0.488
Porcelain aorta	3 (1.2)	4 (1.6)	0.034	1.000
Atrial fibrillation	117 (45.9)	113 (44.3)	0.031	0.794
Frailty	21 (8.2)	26 (10.2)	0.068	0.542
Active malignancy	7 (2.7)	9 (3.5)	0.045	0.804
ProBNP, ng/l	8985 ± 10,700	8068 ± 9584	0.090	0.588
Aortic valve area, cm^2^	0.62 ± 0.19	0.68 ± 0.18	0.310	0.002
Aortic valve gradient, mmHg				
Mean	46 ± 14	46 ± 16	0.125	0.848
Peak	77 ± 22	74 ± 23	0.018	0.160
Mitral regurgitation			0.086	0.459
Moderate	47 (18.4)	45 (17.6)		
Severe	1 (0.4)	1 (0.4)		
SPAP, mmHg			0.062	0.991
31–55	134 (52.5)	128 (50.2)		
> 55	53 (20.8)	52 (20.4)		
Coronary artery disease	93 (36.5)	98 (38.4)	0.040	1.000
Left main stenosis	6 (2.4)	8 (3.1)	0.048	0.791
Number of diseased vessels	0.5 ± 0.8	0.6 ± 0.9	0.064	0.317
Prior pacemaker	24 (9.4)	24 (9.4)	0.000	1.000
Prior PCI	47 (18.4)	42 (16.5)	0.052	0.712
Prior cardiac surgery	22 (8.6)	21 (8.2)	0.014	1.000
Recent myocardial infarction	15 (5.9)	17 (6.7)	0.033	0.850
Recent AHF	78 (30.6)	74 (29.0)	0.034	0.782
NYHA class IV	60 (23.5)	65 (25.5)	0.046	0.707
Urgency of the procedure			0.040	0.992
Urgent	49 (19.2)	49 (19.2)		
Emergency	2 (0.8)	3 (1.2)		
Planned concomitant revascularization	75 (29.4)	13 (5.1)	0.678	< 0.0001
EuroSCORE II, %	8.7 ± 7.9	9.3 ± 8.9	0.076	0.416
STS score, %	4.8 ± 3.9	5.1 ± 4.5	0.052	0.668

Continuous variables are reported as means ± standard and categorical variables as counts and percentages. Clinical variables were defined according to the EuroSCORE II definition criteria

*SAVR* surgical aortic valve replacement, *TAVR* transcatheter aortic valve replacement, *BMI* body mass index, *eGFR* glomerular filtration estimated according to the MDRD equation, *LVEF* left ventricular ejection fraction, Frailty, GSS grades 2–3, *SPAP* systolic pulmonary artery pressure, *PCI* percutaneous coronary intervention, Recent AHF, hospitalization for acute heat failure < 60 days, *NYHA* New York Heart Association

**Fig. 2:**
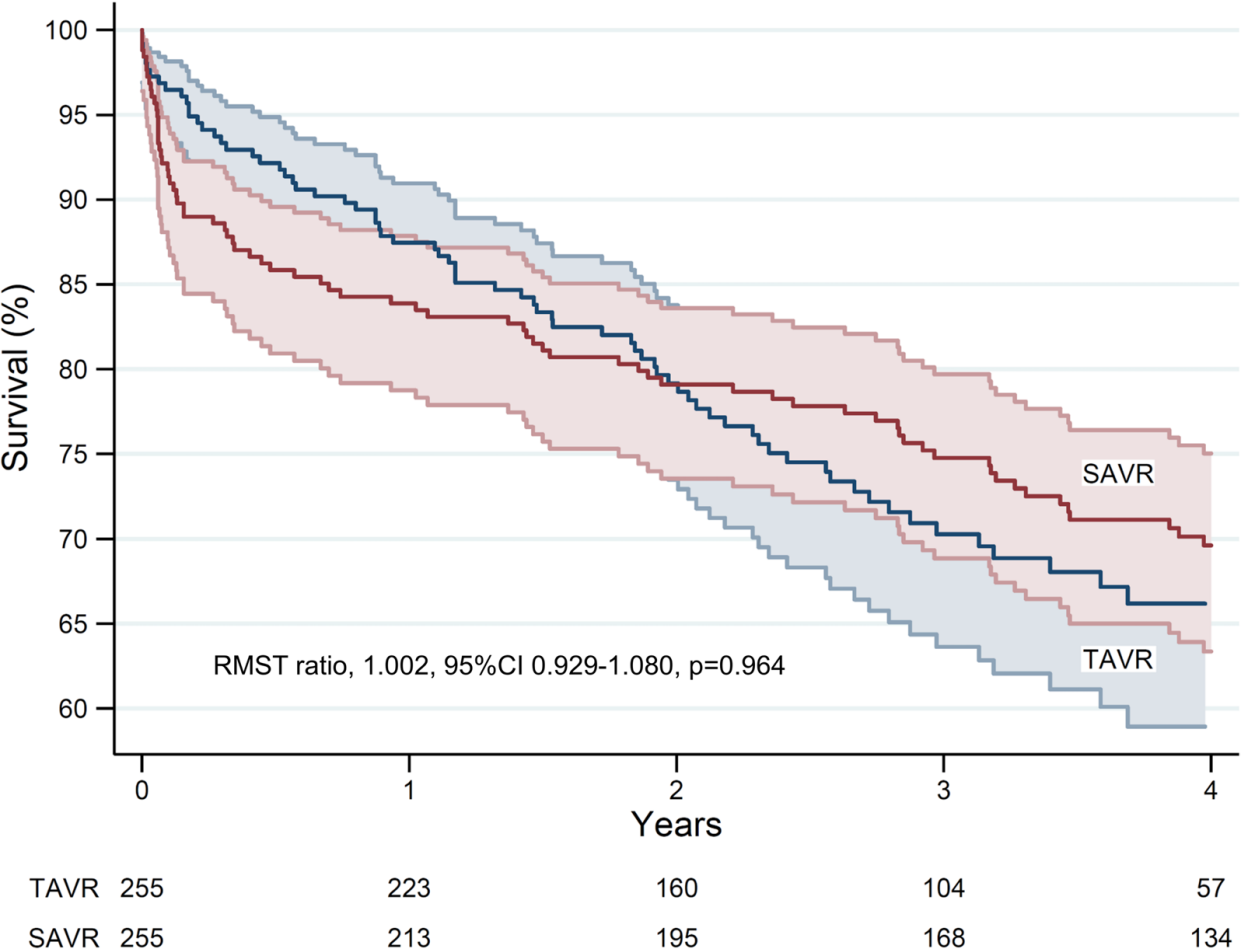
Survival of propensity score matched patients with severe aortic stenosis and reduced left ventricular ejection fraction after surgical and transcatheter aortic valve replacement

Among propensity score matched pairs, SAVR patients had increased rates of bleeding, AKI, blood transfusion, need of mechanical circulatory support and prolonged hospital stay compared to TAVR. TAVR patients had higher rates of vascular complications requiring operation, whereas SAVR patients had increased rates of resternotomy for bleeding (Table 2). Permanent pacemaker implantation rates were more frequent after TAVR. The incidence of postoperative AF was particularly high after SAVR (SAVR 73.7% vs. TAVR 41.6%, *p* < 0.0001). Thirty-day mortality was higher in the SAVR cohort (7.8% vs. 3.1%, *p* = 0.038). One-year and 4-year survival in the TAVR cohort were 87.5% and 65.9% and in the SAVR cohort 83.9% and 69.6% (RMST ratio, 1.002, 95%CI 0.929–1.080, *p* = 0.964) (Fig. 2). During the first 4 years after intervention, the cause of death was of cardiac nature in 69.1% of patients in the SAVR cohort and 51.7% in the TAVR cohort (*p* = 0.043).

**Table 2:** Outcomes in propensity score matched patients with LVEF ≤ 50% undergoing surgical or transcatheter aortic valve replacement

Outcomes	SAVR	TAVR	*p*-value
	(n = 255)	(n = 255)	
Survival, %			
30-day	92.2	96.9	0.038
1-year	84.2	87.7	0.649
4-year	69.6	65.9	0.964
Atrial fibrillation	188 (73.7)	106 (41.6)	< 0.0001
Stroke	12 (4.7)	5 (2.0)	0.143
ECMO and/or IABP	10 (3.9)	0 (0.0)	0.002
Coronary ostium occlusion	0 (0.0)	1 (0.4)	1.000
Aortic damage	3 (1.2)	1 (0.4)	< 0.0001
Vascular complication	4 (1.6)	35 (13.7)	< 0.0001
RBC units transfused	3.6 ± 3.6	0.5 ± 1.2	< 0.0001
E-CABG bleeding grades 2–3	77 (30.6)	7 (2.8)	< 0.0001
Resternotomy for bleeding	18 (7.1)	3 (1.2)	0.001
Acute kidney injury			< 0.0001
Stage 2	12 (4.8)	4 (1.6)	
Stage 3	6 (2.4)	2 (0.8)	
Dialysis	7 (2.7)	2 (0.8)	0.180
Paravalvular regurgitation			0.622
Mild	19 (7.5)	51 (20.0)	
Moderate	0 (0.0)	11 (4.3)	
Severe	1 (0.4)	1 (0.4)	
Permanent pacemaker implantation	9 (3.5)	24 (9.4)	0.009
Hospital stay, days	9.3 ± 6.5	5.4 ± 4.0	< 0.0001

Continuous variables are reported as means ± standard deviation. Categorical variables as counts and percentages

*SAVR* surgical aortic valve replacement, *TAVR* transcatheter aortic valve replacement, Cardiac death, cardiac death within 4 years of index intervention, *ECMO* extracorporeal membrane oxygenation, *IABP* intra-aortic balloon pump; Vascular complication, Major peripheral vascular complication, *RBC* red blood cells, E-CABG bleeding grades 2–3, RBC > 4 units transfused and/or resternotomy for bleeding

## Discussion

This study provides compelling data on the current treatment practice and outcomes of the patients with severe AS and LVEF ≤ 50% in a nationwide setting. The main findings are: (1) early mortality was increased after SAVR; (2) intermediate-term survival was similar after TAVR and SAVR; (3) non-cardiac death was common in this elderly population with multiple co-morbidities.

Patients with LVEF ≤ 50% have poorer prognosis compared to patients with normal systolic function and the prognosis is impaired even after aortic valve operation [4, 16, 17]. In the present study population, only 69.6% of SAVR patients and 65.9% of TAVR patients survived beyond 4-year follow-up. Similarly, in our earlier study [18] recent acute heart failure was associated with worse 30-day and 5-year survival compared to patients with no recent hospitalization for heart failure.

The development of heart failure in AS patients is of multifactorial nature [19–21]. Only 50% of the AS patients with heart failure have reduced LVEF and LVEF reduction in this population is often not caused by AS, but rather myocardial damage due to ischemic heart disease or other cardiomyopathies [22]. Also, the significance of sex, diastolic dysfunction and left bundle branch block are recognized [23]. Still, the ultimate cause of LVEF deterioration in AS remains unresolved. The extent of cardiac damage correlates to worse outcomes [24], even despite still normal LVEF [25–27]. The diastolic dysfunction has already developed when LVEF starts to decrease [28]. Data on diastolic function were not collected in our study population.

The risk for mortality and morbidity increases after surgery with worsening LVEF, and decreased LVEF has been shown being an independent predictor of mortality at 5-year [17, 26]. On the other hand, data from the TVT Registry showed that low-gradient severe AS, rather than the level of baseline LV dysfunction, was associated with 1-year mortality after TAVR [16]. In the present study, at 4-year follow-up the degree of LV dysfunction did not affect survival. Even LVEF < 60% is found to be a risk factor for inferior prognosis [26, 27]. Early intervention may be beneficial for asymptomatic patients with very severe AS [29, 30].

The possible benefits of TAVR over SAVR are unclear for intermediate-risk patients with LVEF ≤ 50% [31, 32]. The procedure type did not affect LVEF recovery in the PARTNER trial including patients with moderate LV dysfunction [33]. LVEF ≤ 50% is associated with an increased risk of sudden cardiac death and all-cause mortality after TAVR, despite LVEF postprocedural improvement [21]. New-onset conduction disturbances and/or the need for a new pacemaker after TAVR are associated with a failure of LVEF recovery after TAVR [34].

Coronary artery revascularization was performed more often during SAVR than with TAVR reflecting the contemporary practice and guidelines. Leaving coronary artery disease untreated during SAVR impairs long-term survival regardless of disease severity [35]. Wolff et al. conclude on their meta-analysis on patients with heart failure with reduced LVEF and coronary disease, that revascularization with either CABG or PCI improves the long-term survival [36]. Recent meta-analysis from Sakurai et al. suggests that patients who underwent TAVR with PCI had a higher all-cause mortality than those with SAVR with CABG [37]. Still, data on concomitant revascularization during TAVR is controversial and scarce. The multi-disciplinary Heart team approach remains imperative for patients with AS and coronary artery disease [37].

## Limitations

The main limitation of this study is its retrospective nature. Second, there may be some degree of interobserver variability in the echocardiographic data and the timing of pre- and perioperative echocardiography varied between the cohorts and institutions. Third, the registry does not capture specific data on the type of aortic stenosis such as high-gradient, low-flow low-gradient, and normal-flow low-gradient AS and an analysis of the subtypes of AS is not feasible. Fourth, the comparison of the study cohorts is based on propensity score matching and its results are potentially biased by unmeasured confounders. Fifth, risks associated with ischemic cardiomyopathy and differences in procedure type related revascularization strategies may affect the results. Finally, the relatively small sample size of this study may affect the reliability of the findings.

## Conclusions

This nationwide registry demonstrated that AS patients with LVEF ≤ 50% have high morbidity and mortality after SAVR and TAVR with no difference in intermediate-term survival between these treatment methods. These findings are in line with previous studies evaluating high-risk patients and patients with LV dysfunction. Further studies on the timing of treatment and treatment pathway choice are needed to optimize the outcomes individually.

## Supplementary Information

**Additional file 1: Fig. S1.** Frequencies of transcatheter (TAVR) and surgical aortic valve replacement (SAVR) along the study period in patients with severe aortic stenosis and reduced left ventricular ejection fraction (LVEF≤50%). **Table S1**. Characteristics of unmatched patients with left ventricular ejection fraction >50% and ≤50% undergoing surgical or transcatheter aortic valve replacement. **Table S2**. Outcomes of unmatched patients with left ventricular ejection fraction >50% and ≤50% after surgical or transcatheter aortic valve replacement. **Table S3**. Left ventricular ejection fraction and NYHA classes of unmatched patients with left ventricular ejection fraction ≤50% undergoing surgical or transcatheter aortic valve replacement. **Table S4**. The effect of baseline left ventricular ejection fraction on survival in unmatched patients with LVEF≤50% undergoing surgical or transcatheter aortic valve replacement.

## Data Availability

The data that support the findings of this study are available from the corresponding author upon reasonable request.

## Abbreviations

AF: Atrial fibrillation
AKI: Acute kidney injury
AS: Aortic stenosis
ECMO: Extracorporeal membrane oxygenation
KDIGO: Kidney disease: improving global outcomes
IABP: Intra-aortic balloon pump
LV: Left ventricle
LVEF: Left ventricular ejection fraction
RBC: Red blood cell
SAVR: Surgical aortic valve replacement
TAVR: Transcatheter aortic valve replacement
