# Supplementary material for: Transcatheter and surgical aortic valve replacement in patients with left ventricular dysfunction
Source: J Cardiothorac Surg. 2022 Dec 18;17:322. doi: 10.1186/s13019-022-02061-9 (PMC9759878; doi:10.1186/s13019-022-02061-9)
Supplement: Supplementary file 1 — Additional file 1: Fig. S1. Frequencies of transcatheter (TAVR) and surgical aortic valve replacement (SAVR) along the study period in patients with severe aortic stenosis and reduced left ventricular ejection fraction (LVEF≤50%). Table S1. Characteristics of unmatched patients with left ventricular ejection fraction >50% and ≤50% undergoing surgical or transcatheter aortic valve replacement. Table S2. Outcomes of unmatched patients with left ventricular ejection fraction >50% and ≤50% after surgical or transcatheter aortic valve replacement. Table S3. Left ventricular ejection fraction and NYHA classes of unmatched patients with left ventricular ejection fraction ≤50% undergoing surgical or transcatheter aortic valve replacement. Table S4. The effect of baseline left ventricular ejection fraction on survival in unmatched patients with LVEF≤50% undergoing surgical or transcatheter aortic valve replacement. [file 13019_2022_2061_MOESM1_ESM.docx]

**Supplementary appendix**

# **Transcatheter and Surgical Aortic Valve Replacement in Patients with Left Ventricular Dysfunction**

Maina P. Jalava et al.

**Supplementary figure S1.** Frequencies of transcatheter (TAVR) and surgical aortic valve replacement (SAVR) along the study period in patients with severe aortic stenosis and reduced left ventricular ejection fraction (LVEF≤50%).

**Supplementary table 1.** Characteristics of unmatched patients with left ventricular ejection fraction >50% and ≤50% undergoing surgical or transcatheter aortic valve replacement.

## Supplementary table 2. Outcomes of unmatched patients with left ventricular ejection fraction >50% and ≤50% after surgical or transcatheter aortic valve replacement.

**Supplementary table 3**. Left ventricular ejection fraction and NYHA classes of unmatched patients with left ventricular ejection fraction ≤50% undergoing surgical or transcatheter aortic valve replacement.

## Supplementary table 4. The effect of baseline left ventricular ejection fraction on survival in unmatched patients with LVEF≤50% undergoing surgical or transcatheter aortic valve replacement.

**Supplementary figure S1.** Frequencies of transcatheter (TAVR) and surgical aortic valve replacement (SAVR) along the study period in patients with severe aortic stenosis and reduced left ventricular ejection fraction (LVEF≤50%).

**
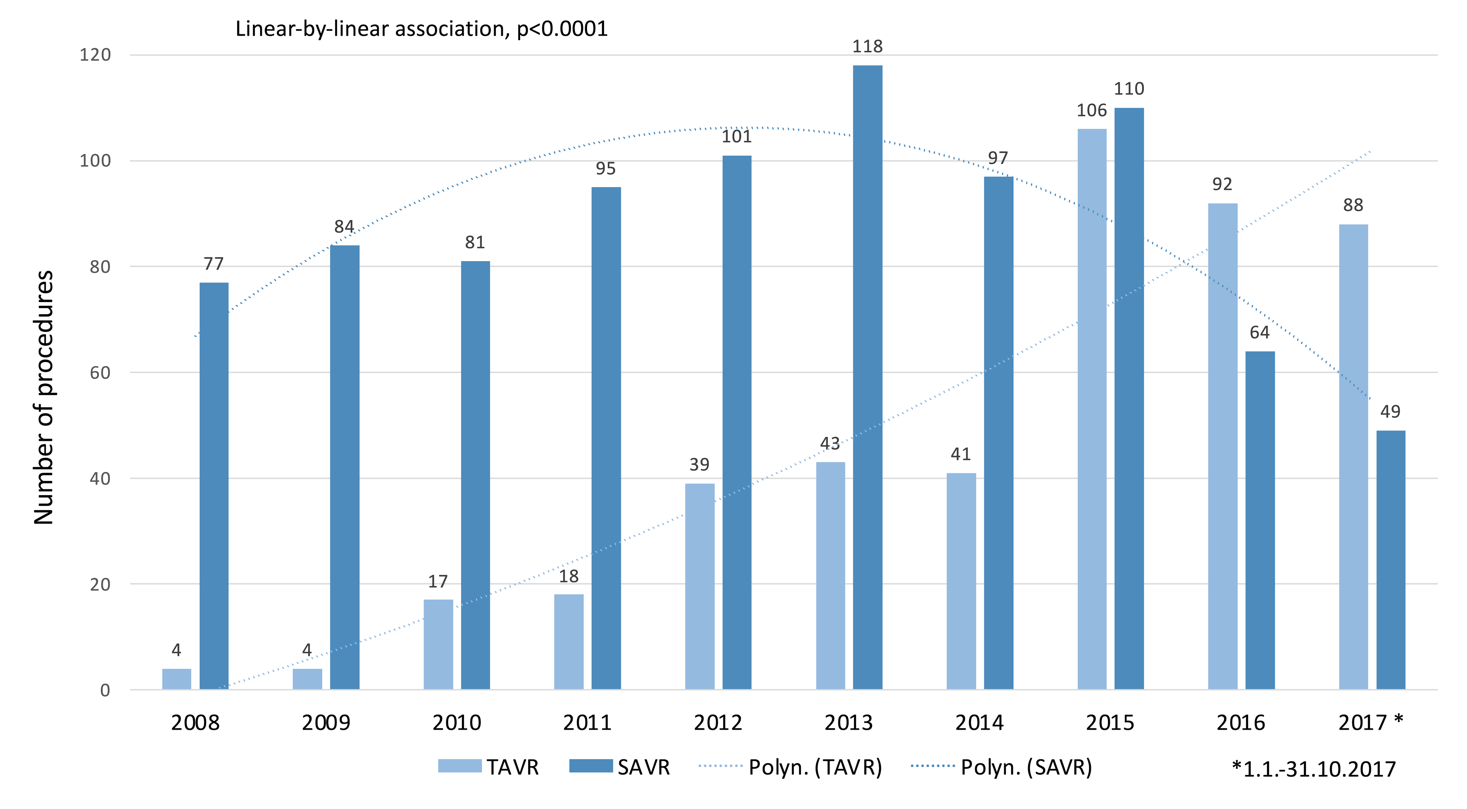
**

**Supplementary table 1.** Characteristics of unmatched patients with left ventricular ejection fraction >50% and ≤50% undergoing surgical or transcatheter aortic valve replacement.

|  | SAVR | |  | TAVR | |  |
| --- | --- | --- | --- | --- | --- | --- |
| Characteristics | LVEF>50%  3344 pts | LVEF≤50%  876 pts | p-value | LVEF>50%  1182 pts | LVEF≤50%  452 pts | p-value |
| Age, years | 75.1±6.5 | 75.1±6.7 | 0.842 | 82.0±6.3 | 80.4±6.8 | <0.0001 |
| Female | 1683 (50.3) | 290 (33.1) | <0.0001 | 738 (62.4) | 182 (40.3) | <0.0001 |
| BMI, kg/m2 | 27.7±4.8 | 27.4±5.0 | 0.012 | 27.3±5.0 | 26.3±4.5 | <0.0001 |
| ProBNP, ng/l | 1388±1865 | 7832±9683 | <0.001 | 3885±10974 | 9463±15823 | <0.001 |
| Aortic valve area, cm^2^ | 0.78±1.70 | 0.72±0.24 | 0.356 | 0.66±0.34 | 0.64±0.21 | 0.230 |
| Aortic valve gradient, mmHg  Peak  Mean | 79±22  49±14 | 71±24  43±15 | <0.001  <0.001 | 80±21  50±15 | 72±22  44±15 | <0.001  <0.001 |
| Anemia | 843 (25.2) | 317 (36.2) | <0.0001 | 506 (42.8) | 248 (54.9) | <0.0001 |
| eGFR, ml/min/1.73m2 | 76.7±21.2 | 72.1±22.8 | <0.0001 | 66.5±22.5 | 62.8±23.1 | 0.001 |
| Dialysis | 9 (0.3) | 4 (0.5) | 0.324 | 11 (0.9) | 7 (1.5) | 0.284 |
| Diabetes | 840 (25.1) | 279 (31.8) | <0.0001 | 317 (26.8) | 137 (30.1) | 0.187 |
| Stroke | 215 (6.4) | 79 (0.0) | 0.007 | 143 (12.1) | 52 (11.5) | 0.741 |
| Atrial fibrillation | 645 (19.3) | 292 (33.3) | <0.0001 | 504 (42.6) | 235 (52.0) | 0.001 |
| Frailty | 79 (2.4) | 27 (3.1) | 0.226 | 182 (15.4) | 76 (16.8) | 0.482 |
| Coronary artery disease | 1450 (43.4) | 468 (53.4) | <0.0001 | 339 (28.7) | 159 (35.2) | 0.011 |
| Left main stenosis | 181 (5.4) | 71 (8.1) | 0.003 | 16 (1.4) | 9 (2.0) | 0.001 |
| Number of diseased vessels | 0.8±1.1 | 1.1±1.2 | <0.0001 | 0.4±0.7 | 0.5±0.8 | 0.018 |
| Prior pacemaker | 117 (3.5) | 53 (6.1) | 0.001 | 94 (8.0) | 76 (16.8) | <0.0001 |
| Prior PCI | 303 (9.1) | 89 (10.2) | 0.319 | 220 (18.6) | 107 (23.7) | 0.022 |
| Prior cardiac surgery | 70 (2.1) | 25 (2.9) | 0.177 | 172 (14.6) | 126 (27.9) | <0.0001 |
| Recent myocardial infarction | 163 (4.9) | 139 (15.9) | <0.0001 | 19 (1.6) | 23 (5.1) | <0.0001 |
| Recent AHF | 189 (5.7) | 299 (34.2) | <0.0001 | 88 (7.4) | 123 (27.3) | <0.0001 |
| NYHA class IV | 184 (5.5) | 254 (29.0) | <0.0001 | 101 (8.5) | 107 (23.7) | <0.0001 |
| Urgency |  |  | <0.0001 |  |  | <0.0001 |
| Urgent | 263 (7.9) | 254 (29.0) |  | 54 (4.6) | 72 (15.9) |  |
| Emergency | 17 (0.5) | 40 (4.6) |  | 1 (0.1) | 5 (1.1) |  |
| SPAP, mmHg |  |  | <0.0001 |  |  | <0.0001 |
| 31-55 | 1156 (34.9) | 370 (42.2) |  | 556 (47.0) | 234 (51.8) |  |
| >55 | 143 (4.3) | 160 (18.3) |  | 148 (12.5) | 101 (22.3) |  |
| Mitral valve regurgitation |  |  | <0.0001 |  |  | <0.0001 |
| Moderate | 134 (4.0) | 109 (12.4) |  | 140 (11.8) | 97 (21.5) |  |
| Severe | 1 (0.1) | 2 (0.2) |  | 6 (0.5) | 10 (2.2) |  |
| Porcelain aorta | 12 (0.4) | 3 (0.3) | 1.0000 | 53 (4.5) | 23 (5.1) | 0.604 |
| Extracardiac arteriopathy | 397 (11.9) | 128 (14.6) | 0.029 | 170 (14.4) | 88 (19.5) | 0.012 |
| Pulmonary disease | 459 (13.7) | 169 (19.3) | <0.0001 | 254 (21.5) | 104 (23.0) | 0.506 |
| Oxygen therapy | 12 (0.4) | 4 (0.5) | 0.756 | 9 (0.8) | 3 (0.7) | 1.000 |
| Active malignancy | 44 (1.3) | 15 (1.7) | 0.374 | 50 (4.2) | 15 (3.3) | 0.399 |
| Planned concomitant revascularization | 1351 (40.4) | 438 (50.0) | <0.0001 | 61 (5.2) | 25 (5.5) | 0.764 |
| EuroSCORE II, % | 3.1±3.0 | 8.5± 9.4 | <0.0001 | 5.5±4.4 | 12.4±11.8 | <0.0001 |
| STS Score, % | 2.6±1.9 | 4.7± 4.8 | <0.0001 | 4.3±2.6 | 5.8±5.0 | <0.0001 |

Continuous variables are reported as mean±standard deviation and categorical variables as counts and percentages. Clinical variables were defined according to the EuroSCORE II definition criteria. SAVR, surgical aortic valve replacement; TAVR, transcatheter aortic valve replacement; LVEF, left ventricular ejection fraction; BMI, body mass index; BNP, B-type natriuretic peptide; PCI, percutaneous coronary intervention; Recent AHF, hospitalization for acute heat failure <60days; NYHA, New York Heart Association; eGFR, glomerular filtration estimated according to the MDRD equation; Frailty, GSS grades 2-3; SPAP, systolic pulmonary artery pressure.

## Supplementary table 2. Outcomes of unmatched patients with left ventricular ejection fraction >50% and ≤50% after surgical or transcatheter aortic valve replacement.

|  | SAVR | |  | TAVR | |  |
| --- | --- | --- | --- | --- | --- | --- |
| Outcomes | LVEF>50%  3344 pts | LVEF≤50%  876 pts | p-value | LVEF>50%  1182 pts | LVEF≤50%  452 pts | p-value |
| Survival, % |  |  | <0.0001 |  |  | <0.0001 |
| 30-day | 97.0 | 94.1 |  | 98.1 | 96.5 |  |
| 1-year | 94.4 | 86.9 |  | 92.9 | 86.7 |  |
| 4-year | 85.2 | 74.5 |  | 69.8 | 62.3 |  |
| Atrial fibrillation † | 1863 (55.7) | 532 (60.7) | 0.008 | 452 (38.2) | 216 (47.8) | <0.0001 |
| Stroke | 121 (3.6) | 38 (4.3) | 0.319 | 27 (2.3) | 11 (2.4) | 0.858 |
| Postop. ECMO and/or IABP | 37 (1.1) | 38 (4.3) | <0.0001 | 1 (0.1) | 0 (0.0) | 1.000 |
| Aortic damage | 20 (0.6) | 10 (1.1) | 0.088 | 9 (0.8) | 3 (0.7) | 1.000 |
| Vascular complication | 46 (1.4) | 20 (2.3) | 0.054 | 30 (2.5) | 13 (2.9) | 0.517 |
| RBC, units transfused | 2.8±3.7 | 3.5±4.2 | <0.0001 | 0.5±1.6 | 0.5±1.3 | 0.731 |
| >4 units transfused | 652 (19.8) | 246 (28.4) | <0.0001 | 37 (3.2) | 13 (2.9) | 0.816 |
| E-CABG bleeding grades 2-3 | 739 (22.4) | 265 (30.6) | <0.0001 | 39 (3.3) | 16 (3.6) | 0.781 |
| Resternotomy for bleeding | 266 (8.0) | 72 (8.2) | 0.797 | 6 (0.5) | 4 (0.9) | 0.477 |
| Acute kidney injury |  |  | <0.0001 |  |  | 0.118 |
| Stage 2 | 81 (2.4) | 42 (4.9) |  | 14 (1.2) | 8 (1.8) |  |
| Stage 3 | 87 (2.6) | 33 (3.8) |  | 6 (0.5) | 7 (1.6) |  |
| Dialysis |  |  | 0.115 |  |  | 0.009 |
| Temporary | 58 (1.7) | 21 (2.4) |  | 4 (0.3) | 8 (1.8) |  |
| Permanent | 18 (0.5) | 9 (1.0) |  | 8 (0.7) | 2 (0.4) |  |
| Permanent pacemaker implantation | 131 (3.9) | 35 (4.0) | 0.916 | 116 (9.8) | 42 (9.3) | 0.760 |
| Paravalvular regurgitation |  |  | 0.085 |  |  | 0.239 |
| Mild | 166 (5.0) | 50 (5.7) |  | 274 (23.2) | 90 (19.9) |  |
| Moderate | 18 (0.5) | 1 (0.1) |  | 49 (4.1) | 20 (4.4) |  |
| Severe | 5 (0.1) | 4 (0.5) |  | 1 (0.1) | 2 (0.4) |  |
| Hospital stay, days | 8.1±6.2 | 9.1±7.0 | <0.0001 | 5.4±4.6 | 5.5±4.5 | 0.123 |

Continuous variables are reported as means±standard and categorical variables as counts and percentages. SAVR, surgical aortic valve replacement; TAVR, transcatheter aortic valve replacement; LVEF, left ventricular ejection fraction; ECMO, extracorporeal membranous oxygenation; IABP, intra-aortic balloon pump; Aortic damage, aortic dissection or rupture; Vascular complication, Major peripheral vascular complication; RBC, red blood cell.

† Any postoperative atrial fibrillation during index hospitalization

## Supplementary table 3. Left ventricular ejection fraction and NYHA classes of unmatched patients with left ventricular ejection fraction ≤50% undergoing surgical or transcatheter aortic valve replacement.

|  | SAVR | TAVR | p-value |
| --- | --- | --- | --- |
|  | 876 pts | 452 pts |  |
| LVEF classes, n (%) |  |  | 0.117 |
| 30-50% | 760 (86.8) | 373 (82.5) |  |
| 21-30% | 92 (10.5) | 62 (13.7) |  |
| <21% | 24 (2.7) | 17 (3.8) |  |
| NYHA classes |  |  | <0.0001 |
| I | 6 (0.7) | 2 (0.4) |  |
| II | 166 (18.9) | 50 (11.1) |  |
| III | 450 (51.4) | 293 (64.8) |  |
| IV | 254 (29.0) | 107 (23.7) |  |

SAVR, surgical aortic valve replacement; TAVR, transcatheter aortic valve replacement; LVEF, left ventricular ejection fraction; NYHA, New York Heart Association.

## Supplementary table 4. The effect of baseline left ventricular ejection fraction on survival in unmatched patients with LVEF≤50% undergoing surgical or transcatheter aortic valve replacement.

|  | SAVR | | p-value | TAVR | | p-value |
| --- | --- | --- | --- | --- | --- | --- |
| LVEF | 30-50% | <30% |  | 30-50% | <30% |  |
| Survival, % |  |  | 0.641 |  |  | 0.514 |
| 1-year | 87.0 | 85.3 |  | 89.3 | 75.9 |  |
| 2-year | 84.0 | 81.8 |  | 80.0 | 71.6 |  |
| 3-year | 78.9 | 79.9 |  | 71.0 | 64.3 |  |
| 4-year | 74.2 | 78.0 |  | 62.1 | 62.4 |  |

Survival rates were estimated with the Kaplan-Meier method. SAVR, surgical aortic valve replacement; TAVR, transcatheter aortic valve replacement; LVEF, left ventricular ejection fraction.
